# Expression of mGluR5 in Pediatric Hodgkin and Non-Hodgkin lymphoma—A Comparative Analysis of Immunohistochemical and Clinical Findings Regarding the Association between Tumor and Paraneoplastic Neurological Disease

**DOI:** 10.3390/cancers16132452

**Published:** 2024-07-04

**Authors:** Ingeborg Viezens, Ellen Knierim, Hedwig E. Deubzer, Kathrin Hauptmann, Jessica Fassbender, Susanne Morales-Gonzalez, Angela M. Kaindl, Markus Schuelke, Marc Nikolaus

**Affiliations:** 1 NeuroCure Cluster of Excellence, Charité-Universitätsmedizin Berlin, 10117 Berlin, Germany; ingeborg.viezens@charite.de (I.V.); ellen.knierim@charite.de (E.K.); jessica.fassbender@charite.de (J.F.); susanne.morales-gonzalez@charite.de (S.M.-G.); markus.schuelke@charite.de (M.S.); 2 Department of Pediatric Neurology, Charité-Universitätsmedizin Berlin, 10117 Berlin, Germany; angela.kaindl@charite.de; 3 DRK Kliniken Westend, Klinik für Kinder- und Jugendmedizin, 14050 Berlin, Germany; 4 Department of Pediatric Oncology/Hematology, Charité-Universitätsmedizin Berlin, 10117 Berlin, Germany; hedwig.deubzer@charite.de; 5 Experimental and Clinical Research Center (ECRC), Charité and Max-Delbrück-Center of Molecular Medicine, Helmholtz Association, 13125 Berlin, Germany; 6 Institute of Pathology, Charité-Universitätsmedizin Berlin, 10117 Berlin, Germany; kathrin.haupmann@charite.de; 7 Center for Chronically Sick Children, Charité-Universitätsmedizin Berlin, 10117 Berlin, Germany; 8 Institute for Cell and Neurobiology, Charité-Universitätsmedizin Berlin, 10117 Berlin, Germany

**Keywords:** pediatric Hodgkin lymphoma, autoimmune encephalitis, Ophelia syndrome, mGluR5, paraneoplastic neurological syndromes, EBV-positive HL

## Abstract

**Simple Summary:**

Autoantibodies against the metabotropic glutamate receptor 5 (mGluR5) have been linked to Ophelia syndrome, a combination of limbic encephalitis and Hodgkin lymphoma (HL). We studied mGluR5 expression in 57 pediatric HL and NHL by immunohistochemistry to explore the relationship between mGluR5 antibody formation and HL. All lymphoma tissues displayed mGluR5 staining, especially HL Reed–Sternberg cells. There was no staining in age-matched healthy lymph nodes, and we did not find *GRM5* transcripts in RNA-sequencing data from normal lymphocytes. Lower mGluR5 levels in HL correlated with younger patients and EBV-positive tumors. Cases of paraneoplastic and neurological symptoms were found exclusively in the HL cohort. The frequent presence of mGluR5 in lymphoma tissue suggests a possible role in tumor development. This finding is in line with reports that glutamatergic signaling affects the outcome in other cancers. However, further studies in larger cohorts are needed to evaluate the impact of mGluR5 on HL severity.

**Abstract:**

Autoantibodies targeting the neuronal antigen metabotropic glutamate receptor 5 (mGluR5) have been identified in patients with Ophelia syndrome, which describes a co-occurrence of paraneoplastic limbic encephalitis and Hodgkin lymphoma (HL). Little data exist regarding frequency and function of mGluR5 in HL and its potential role in causing seropositive paraneoplastic disease. We studied a representative cohort of pediatric HL and NHL patients (n = 57) using immunohistochemistry and fluorescence staining to investigate mGluR5 expression. All lymphoma tissues displayed positive mGluR5 staining, with focus on Hodgkin–Reed–Sternberg (H-RS) cells. We did not detect any mGluR5 staining in tumor-free lymph nodes, which is consistent with the absence of *GRM5* transcripts in RNA-sequencing data from non-malignant B and T cells. The frequent presence in pediatric lymphoma falls in line with reports of mGluR5 expression and associated tumor progression in other malignancies. We tested for correlation with clinical features, focusing on disease progression and neurological symptoms. Low mGluR5 expression in H-RS cells correlated with young patient age (<15 years) and positive histology for EBV infection. Paraneoplastic or neurological symptoms were found exclusively in HL patients. While an impact of mGluR5 on HL severity remains possible, a prognostic value of mGluR5 expression levels requires further investigation.

## 1. Introduction

The term Ophelia syndrome describes the combination of paraneoplastic limbic encephalitis (PLE) with Hodgkin lymphoma (HL). The syndrome was first reported in 1982 by Dr. Ian Carr who suggested “circulating molecule-like neurotransmitters produced by the neoplasm” as a potential cause of memory loss and hallucinations [1]. In 2011 Lancaster et al. discovered autoantibodies against the metabotropic glutamate receptor 5 (mGluR5) in the serum and cerebrospinal fluid (CSF) of two patients with Ophelia syndrome [2]. The receptor, encoded by *GRM5*, belongs to a group of glutamate receptors normally found in both the peripheral and central nervous system. This group subdivides into **(i)** mGluR1 and mGluR5, **(ii)** mGluR2 and mGluR3 and **(iii)** mGluR4 and mGluR6–8 [3]. These receptors are known to be involved in a variety of neurological functions including learning, memory, pain perception and anxiety [4]. Antibodies against three of these receptors (mGluR1, 2 and 5) have been identified in patients with paraneoplastic neurological disorders [2,5,6]. In recent years, metabotropic glutamate signaling has been implicated in tumor regulation and in cancer development of neuronal and non-neuronal origin [7]. Several in vitro studies have specifically implicated mGluR5 in promoting tumor progression and formation [8,9,10,11,12]. Further experiments with mGluR5 antibodies from patients with autoimmune encephalitis demonstrated their pathogenicity by showing a reversible reduction in receptor density in animal models [13]. Our group was the first to show results compatible with the hypothesis of mGluR5 as an aberrant onconeural cell surface antigen, possibly triggering an autoimmune response due to its expression in HL tumor cells. Inspired by a case of pediatric Ophelia syndrome with severe mGluR5 antibody encephalitis, we provided experimental evidence of heterogeneous mGluR5/*GRM5* expression in different HL cell lines and in tumor tissue of the index patient [8].

In the present study, we aimed **(i)** to confirm previous findings of mGluR5 in cultured HL cells now in actual lymphoma tissue using a larger cohort of pediatric HL cases, **(ii)** to examine the frequency and localization of the antigen and **(iii)** to investigate a possible association of mGluR5 expression in tumor tissue with clinical features and disease progression. In particular, we focused on the prevalence of neurological symptoms and a history of autoimmune or paraneoplastic disease. Furthermore, we sought to determine whether mGluR5 is found exclusively or predominantly in HL, as most cases with paraneoplastic mGluR5 antibodies were diagnosed with this lymphoma subtype [14]. A control group of pediatric non-Hodgkin lymphoma (NHL) patients and additional tumor-free lymph node samples were therefore used for comparison. Our study aims to improve the understanding of the complex interplay between HL, autoimmune responses and its associations with rare paraneoplastic neurological syndromes. This understanding may contribute to the development of new diagnostic and therapeutic approaches.

## 2. Materials and Methods

### 2.1. Patients and Tissue Samples

We established clinical cohorts of pediatric HL and NHL cases by reviewing all patients treated for lymphoma between 2008 and 2021 at the Department of Pediatric Hematology and Oncology of the Charité—Universitätsmedizin Berlin (HL n = 56, NHL n = 66). Formalin-fixed, paraffin-embedded, 3 µm thick tumor tissue sections were obtained from the Department of Pathology of the Charité—Universitätsmedizin Berlin for all cases with available biopsy material (n = 57; HL n = 29, NHL n = 28). All HL samples were derived from lymph node tissue. The NHL sections represent a heterogeneous histologic background (respiratory tract mucosa, lymph node, bone marrow, tonsil, skeletal muscle and ovary). Three tissue samples were obtained from patients with a lymphoma relapse. We reviewed the medical histories of all included cases for clinical parameters, symptoms and disease course with a focus on neurology and autoimmunity. As additional controls, we acquired age-matched formalin-fixed, paraffin-embedded lymph node tissue sections of n = 6 children with the histopathologic diagnosis of tumor-free and healthy lymph node tissue or, in one case, lymphadenitis due to massive cholestasis. Most of the control samples are hepatic lymph nodes (n = 4) coming from liver transplantation operations due to biliary atresia. The remaining controls were obtained from the paracaval or cervical area after surgery for nephroblastoma or rhabdomyosarcoma. The study was conducted in accordance with the tenets of the Declaration of Helsinki. It involved the use of material of human origin. The study was approved by the Ethics Committee of Charité—Universitätsmedizin Berlin (EA2/121/17). 

### 2.2. Immunohistochemistry with DAB for CD30 and mGluR5 on HL and NHL Tissue

For DAB staining we used the previously described formalin-fixed, paraffin-embedded sections from HL, NHL and control patients. We performed staining with anti-mGluR5 primary antibody (1:200, Cat# PA5-33823 Invitrogen Waltham, MA, USA) on tissue sections from each patient. In addition, all pathologically confirmed CD30-positive tumor tissue sections (HL n = 29; NHL n = 5) were stained with anti-CD30 primary antibody (1:20, Ber-H2, Cat# M0751, Dako, Jena, Germany). We used an automated slide staining system (BenchMark XT, Ventana Medical Systems, Tucson, AZ, USA), performed heat-induced epitope retrieval (Cell Conditioning 1, Ventana) and additional hematoxylin staining for cell visualization and secondary staining with an automated DAB staining kit (Ultra View TM Universal DAB-Kit, Roche Diagnostics (Indianapolis, IN, USA)) were used for all sections.

### 2.3. Analysis of RNA-Sequencing Data of Lymphocytes 

To study the expression of *GRM5* in non-malignant lymphatic cells as controls for the lymphoma tissue experiments, we re-analyzed the following publicly available RNA-sequencing datasets of isolated human B and T cells published in the Gene Expression Omnibus repository (https://www.ncbi.nlm.nih.gov/geo/, accessed on 7 June 2024) of the NCBI (accession numbers: GSE74102, GSE119234, GSE186010). The FASTQ files were aligned to the human reference sequence (GRCh38.p13) using STAR v7.2.10b and quantification was done with the StringTie v2.2.1 program on a high-performance server running 160 cores. As a quantitative measure of gene abundance, we used the FPKM values (fragments per kilobase of transcript per million mapped reads). *GRM5* transcripts were absent from normal regulatory T-cells (GSE74102) that had been extracted from normal lymph nodes (n = 10) and from follicular lymphomas (n = 12). In the normal B-cell population, *GRM5* was only expressed in memory B-cells at very low levels (FPKM = 0.179 [SD 0.412]). It was absent in B-cells from the germinal centers, in naïve B-cells and in unswitched memory B-cells.

### 2.4. Immunofluorescence Co-Staining on Tumor Tissue of HL and NHL Patients

We carried out immunofluorescence staining with primary antibodies against CD30 and mGluR5 on the formalin-fixed tumor tissue sections. For all pathologically confirmed CD30-positive lymphoma cases, we performed a double staining. The remaining CD30-negative tumor samples (NHL n = 23) were stained with the anti-mGluR5 primary antibody only. In preparation for staining, we deparaffinized the tumor sections and performed standardized heat-antigen retrieval (Antigen Unmasking Solution, Citric Acid Based, H-3300, Vector Laboratories (Newark, CA, USA)). For all washing steps and dilution of antibodies we used 1× PBS. The sections were stored in a humid chamber during all incubation steps and blocked for 45 min at RT using normal goat serum with bovine serum albumin. We incubated the tissues overnight at 4 °C with anti-CD30 primary antibody (1:10, monoclonal mouse anti-human, Ber-H2, Cat# M0751, Dako, Jena, Germany) and/or anti-mGluR5 primary antibody (1:200, polyclonal rabbit anti-human IgG, Cat# PA5-33823, Invitrogen Waltham, MA, USA). We used fluorescence-labeled conjugated antibodies for secondary staining and incubated the sections in the dark at RT for 2 h (mGluR5: 1:500, goat anti-rabbit IgG Alexa-Fluor-488, Cat# A-32731, Invitrogen, Waltham, MA, USA; CD30: 1:500, goat anti-mouse IgG1 Alexa-Fluor-568, Cat# A-21124, Invitrogen, Waltham, MA, USA). We applied 4′,6-diamidino-2-phenylindole for nuclei staining (DAPI; 1:1000; Cat# D1306, Invitrogen) and used Polyvinyl alcohol (Mowiol 4-88, Carl Roth, Germany) for fixation. 

For image acquisition we used a THUNDER Imager DMi8 with a Leica DFC9000 GT camera and the LAS(X) software (Leica Application Suite (X), Version 3.7.4.23463, Leica Microsystems, Wetzlar, Germany). The imaging parameters were kept constant for all sections and recordings. We used far-red fluorescence as a control channel to analyze autofluorescence, known to occur in lymphoma tissues [15]. Prior to fluorescence image recording, we evaluated the anti-CD30 DAB-stained sections to identify regions with high numbers of CD30-positive cells to localize tumor infiltration. We recorded at least three 2 × 2 tile-scan images per case with 16-bit resolution using an original objective lens magnification of ×20, using all four fluorescent channels. Additionally, we recorded images at ×100 objective lens magnification to better visualize subcellular staining. All images and metadata were saved in the *.lif format and are available upon request.

### 2.5. Automated Image Analysis of Immunofluorescence Co-Staining 

To analyze the colocalization and staining patterns of CD30 and mGluR5 on the fluorescence images, we used the Fiji/ImageJ v.2.3.0/1.53 software and programmed an automated algorithm (macro), of which the code is available in the Supplementary Materials. We analyzed three images each from separate tile scans per lymphoma case. The selection criteria for the images were **(i)** highest amount of DAPI stained nuclei over the whole picture frame and **(ii)** good quality of the control channel image to evaluate and filter for possible autofluorescence. Using binary masks by thresholding (pixel value 0 or 255; 8-bit) to visualize and measure the staining pattern in a standardized way, we calculated the total cell count, the percentage of CD30-positive cells, as well as the percentage of double positive cells (CD30 AND mGluR5) per image. The parameters and sequence of image analysis (contrast enhancement, subtraction, ROI analysis, introduction of a Gaussian blur filter, etc.) were kept constant for all images, except for background subtraction. We adjusted the rolling ball radius (norm = 100; adjustment 50 or 500) in a few images with low image quality to improve segmentation of actual staining and to reduce apparent false positive signals due to background noise.

### 2.6. Statistical Analysis

Image and statistical analyses were conducted with Fiji/ImageJ v.2.3.0/1.53 and IBM SPSS Statistics v26 (New York, NY, USA). A *p* value of < 0.05 was considered to indicate statistical significance. Quantitative experimental data are presented as the means ± SD of results that had been obtained from at least three independent measurements. We used the t-test for independent samples to calculate the significance of differences in CD30 expression and in CD30 dependent mGluR5 expression between individual tumors. Due to a small test population of below 30 samples, we tested for normal distribution using the Shapiro Wilk and Kolmogorov-Smirnov tests to confirm the required t-test conditions.

To analyze correlation between clinical parameters and experimental findings, we performed the Chi-square, Fishers exact, or Mann–Whitney-U test. To evaluate possible correlation, we used Cramers V and Pearsons R.

## 3. Results

### 3.1. Cohort Description: Diagnosis and Course of Disease in Representative Groups of Children with HL and NHL

We examined the general patient characteristics and individual histories to gain an overview of the composition of our cohort of pediatric lymphoma cases. Table 1 shows the results for sex, age, staging and disease course for the HL and NHL groups. The distribution of histologic subtypes can be found in the Supplemental Figure S1.

In addition, the most frequently documented symptoms were lymphadenopathy, pain and dyspnea due to tumor compression. Fever before diagnosis was described in n = 3 patients with HL and in n = 2 children with NHL. All children were treated according to the EuroNET-PHL-C2 study for HL or the BFM registries for NHL. All patients received first line chemotherapy. The HL cohort divides into three treatment arms reflecting the severity of the disease at the time of diagnosis, with advanced and intermediate stage HL each comprising 41% of patients. Radiation was used in the treatment of primary HL only if the patients had responded inadequately to chemotherapy (n = 5). In the NHL cohort, radiation was used in n = 2 patients. We found that three patients with HL were diagnosed with conditions, classified as paraneoplastic by their treating physicians: one girl presented with a clinical depression 6 months prior to HL diagnosis, one boy developed immune thrombocytopenia (ITP) 11 months prior to the tumor diagnosis and the third child was the index patient with mGluR5 antibody encephalitis (Ophelia syndrome).

#### 3.1.1. HL Patients with Neurologic Symptoms and Paraneoplastic Manifestations

To evaluate lymphoma cases for their neurologic features, we reviewed symptoms, previous diseases and specific diagnostic procedures recorded in the patients’ histories. The results are shown in Table 1. The aim was to study the prevalence of neurological abnormalities in general and to evaluate whether signs of paraneoplastic neurological disease or of mild phenotypes without full manifestation might have been missed. We searched for documented neurological symptoms that could not be explained by tumor infiltration and found four cases of HL. In the NHL cohort, we only saw patients with focal neurological symptoms or with headache related to a respective tumor infiltration of the central nervous system.

**Patient 1** (female, 17 y/o, stage IV B) developed a depression 6 months before the diagnosis of HL, which was officially classified as paraneoplastic. Depression was the first tumor-related symptom followed by lymphadenopathy and B symptoms. All neurological diagnostic procedures such as EEG, cMRI and examination of CSF and blood for autoantibodies, including IF staining, were without pathological findings.

**Patient 2** (female, 16 y/o, stage II A) was diagnosed with a depression 2 months before the diagnosis of HL. Symptoms included a depressed mood, emotional instability, irritability and vertigo. Other lymphoma-related symptoms (lymphadenopathy, bone and joint pain, hair loss) were noted 10 months earlier. No EEG, cMRI or CSF analyses were initiated in this case. The depression was described as ongoing after treatment for HL.

**Patient 3** (male, 16 y/o, stage III A) was the case of mGluR5 antibody encephalitis presenting as Ophelia syndrome. The neurological symptoms started 27 months before the diagnosis of HL, and included headache, visual and auditory verbal hallucinations, photophobia, aggression, fatigue and depersonalization. The symptoms could be reduced by immunotherapy but were still detectable at the time of the lymphoma diagnosis. Despite regular screening for HL following the identification of mGluR5 antibodies in blood and CSF, the tumor was found at an advanced stage. An EEG showed pathologic results during the period of encephalitis.

**Patient 4** (male, 16 y/o, stage IV B) presented with a fever before the diagnosis of HL. During the first two days of chemotherapy, he developed additional visual and auditory hallucinations and became disoriented. The treating physicians diagnosed a prednisolone-induced psychosis, after excluding Herpes simplex encephalitis or CNS tumor infiltration. A cMRI and basic CSF analysis were normal, and screening for autoantibodies was not initiated. An EEG performed one day after the diagnosis of HL, but before the onset of hallucinations and chemotherapy showed localized groups of slow waves in the right frontal region, indicating a possible local brain pathology or unspecified encephalopathy [8].

None of the children with neurological symptoms (n = 10) had a recorded history of neurological or psychiatric disease. In the remaining group of lymphoma patients, we found one case of traumatic brain injury and multiple intracerebral hemorrhages (HL), one child with motor tics (HL) and one patient with Louis–Bar syndrome (NHL). In terms of diagnostic procedures, 18% (7/40) of the EEGs were classified as abnormal. Two cases belonged to patients without neurological symptoms, CNS infiltration or correlating pathology on cMRI. One EEG, coming from an HL patient (female, 11 years, stage II A) showed an intermittent bifrontal dysfunction, indicating possible epileptic potentials. A boy with T-lymphoblastic lymphoma (T-LBL) presented with a right posterior regional dysfunction. All cMRIs with pathological results (HL n = 1, NHL n = 7) showed obvious tumor infiltration or included signs of previously diagnosed disease. CSF analysis was pathological only in the patient with Ophelia syndrome and in the NHL patients with CNS infiltration (n = 6). CSF antibody screening was performed in two cases of HL.

#### 3.1.2. Histopathological Spectrum of Entities and Association with EBV in Our Cohort

To understand the composition and analyze comparability of the biopsy samples, we analyzed the available histologic and immunohistochemistry data.

The HL cohort (n = 29) included 28 samples of classic HL (cHL) and one sample of nodular lymphocyte-predominant HL (NLPHL). The cHL samples can be further differentiated into histologic categories. They divided into the subtypes of nodular sclerosis (n = 20) and mixed cellularity (n = 8). The lymphocyte-rich and lymphocyte-depleted variants were not represented in our cohort. All cHL showed obligatory expression of tumor necrosis factor receptor 8 (CD30) in the Hodgkin and Reed–Sternberg tumor cells (H-RS cells). Positive staining for CD15, a frequently overexpressed antigen in HL cells, was absent in only one case of mixed cellularity cHL. One cHL with nodular sclerosis subtype is described to be of rare T-cell differentiation, identified by missing expression of the transcription factor PAX-5 and positive staining for granzyme B in the H-RS cells. Tumor cells of the nodular lymphocyte-predominant HL (NLPHL) were described as positive for CD15 and CD30 and additionally expressed B-cell lymphoma 6 (BCL6). The tumor milieu of all HL included cells with positive staining for CD3 or CD20, indicating the presence of T and B lymphocytes.

The NHL cohort (n = 28) comprised 18 cases of B-cell lymphoma (18/28, 64%), including Burkitt lymphoma (n = 13), diffuse large B-cell lymphoma (DLBCL; n = 2) and one case each of primal mediastinal B-cell lymphoma (PMBCL), follicular lymphoma and unspecified mature B-cell lymphoma. T-cell lymphomas in the NHL cohort (10/28, 35%) included the entities anaplastic large cell lymphoma (ALCL; n = 3) and T-lymphoblastic lymphoma (T-LBL; n = 7). CD30 expression was detected in n = 5 samples (ALCL n = 3, PMBCL n = 1 and DLCB n = 1).

The histologic association of all HL with Ebstein-Barr Virus (EBV) was routinely evaluated by testing for expression of the viral protein latent membrane protein-1 (LMP-1), indicating latent infection. Expression in H-RS cells was documented in n = 6 cases of HL (6/29, 21%). These children were predominantly male (5/6, 83%) and most cases (4/6, 66%) had the histologic subtype cHL of mixed cellularity. The mean age was on average five years younger in the EBV-positive HL cohort than in the EBV-negative cases (10.5 vs. 15.1 years at time of diagnosis). Positive serology for EBV, indicating prior exposure to the virus, was found in 15/29 (52%) HL patients; 6/15 (40%) subsequently developed EBV-positive lymphoma. Among NHL samples, 19/28 (68%) were tested for LMP-1 expression. One case of Burkitt’s lymphoma (female, 12 y/o) was EBV-positive. Positive EBV serology was detected in 15/27 (55%) children with NHL.

### 3.2. Staining Results Indicating Frequent Expression of mGluR5 in Pediatric Lymphoma Tissue

Using DAB staining, we were able to confirm CD30 expression in all tissues with previously described positivity for the receptor (n = 34). We used these results to validate the staining protocol and tissue quality. When evaluating the sections stained with the mGluR5 antibody, we detected DAB signal in all lymphoma samples, but noted considerable differences in staining patterns and intensities between tissues. We did not see any cells with expression of mGluR5 in the control samples coming from tumor-free lymph nodes.

The CD30 staining allowed us to localize areas of tumor infiltration in the biopsy material, as CD30 is an obligatory antigen on H-RS cells and an optional marker for NHL tumor cells. The CD30-positive cells in cHL had the typical morphology of H-RS cells and were present in all samples. In NLPHL, an HL entity without H-RS cells, CD30 expression was visible in the corresponding tumor cells, called lymphocyte-predominant cells (LP cells). The CD30-positive NHL showed a staining pattern comparable to HL in case of DLBCL and PMBCL with individually stained tumor cells with larger cell bodies. The ALCL samples contained large populations of homogeneous large sized cells, positive for CD30. 

**Figure 1:**
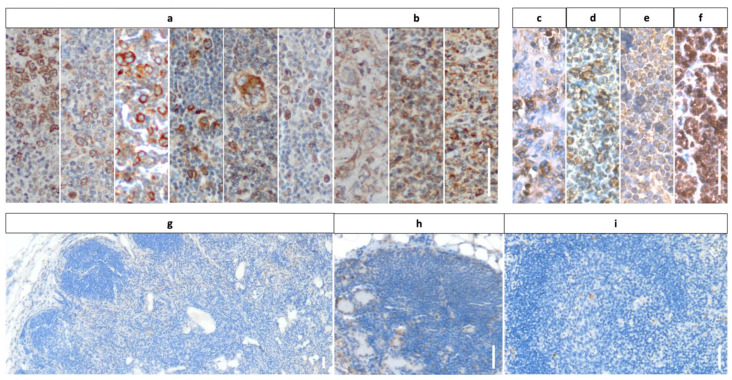
DAB staining of the images of HL (**a**,**b**), NHL (**c**–**f**) and control (**g**–**i**) tissues with mGluR5 antibody shows positive cells in the lymphoma sections and no specific staining in tumor free lymph nodes. (**a**) Examples of HL sections with defined positive tumor cells corresponding in morphology and localization to CD30+ H-RS cells, suggesting elevated expression of mGluR5 in H-RS cells. (**b**) HL sections without individually highlighted tumor cells but with a more homogenous staining pattern. (**c**–**f**) Examples of NHL with mGluR5+ cells and differing intensities between histological subtypes; (**c**) ALCL (**d**) DLBCL (**e**) Burkitt lymphoma (**f**) T-LBL. (**g**–**i**) Healthy lymph node tissues from three individual children do not show any positive staining for mGluR5 in lymphocytes. (**a**–**f**) Original objective lens magnification was ×20 for all lymphoma images; ×5 for (**g**) and ×10 for (**h**,**i**). The scale bar indicates 100 μm in all images.

When analyzing the mGluR5 results for HL tissues, we saw two groups of staining patterns, examples of which are shown in Figure 1a,b. Most HL sections contained individual cells with increased and contrasting staining compared to the surroundings. The cells resembled the previously described CD30-positive H-RS tumor cells in shape, size and localization (Figure 1a). In the minority of HL cases, we found an almost homogeneous DAB staining signal covering the entire cell population including the tumor environment (Figure 1b). Here, the staining results for mGluR5 did not overlap with the corresponding CD30-stained sections, which contained clearly distinguishable H-RS cells. The mGluR5 staining signal in NHL sections (Figure 1c–f) was visible in all cases and we observed variations in intensity and staining pattern in between samples. These variations did not seem to correlate with the lymphoma subtype. 

The DAB staining with mGluR5 antibody of tumor-free pediatric lymph node controls (n = 6) did not show any positive staining. In particular, lymphocytes in the cortex and follicles displayed no trace of mGluR5 in any of the samples. Minor unspecific staining was observed in erythrocyte-containing macrophages and lymphatic sinuses of the medulla (Figure 1g–i).

### 3.3. RNA-Sequencing: No Relevant Expression of GRM5 in Non-Malignant Lymphocytes 

In addition to the immunohistochemical control staining, which indicated the absence of mGluR5 in healthy lymphatic cells, we studied gene expression levels of *GRM5* in lymphocytes via re-analysis of published RNA-sequencing data.

The expression profiles of resident lymph node B cell populations of n = 5 healthy adults were analyzed, including **(i)** germinal center B cells, **(ii)** naïve B cells, **(iii)** unswitched memory B cells and **(iv)** memory B cells. *GRM5* was only expressed in n = 3 memory B cell populations at irrelevant to very low levels. The following analysis of human memory B-cells isolated from lung, lung-draining lymph nodes and peripheral blood mononuclear cells confirmed the results of inconsistent and very low expression of GRM5 in memory B cells in 3 of 13 samples next to missing expression in the remaining samples.

We also did not find any *GRM5* transcripts in regulatory T cells that had been isolated from normal lymph nodes (n = 10), follicular lymphoma lymph nodes (n = 12) and reactive lymph nodes (n = 5).

### 3.4. Immunofluorescence Co-Staining Analysis and Clinical Associations

**Figure 2:**
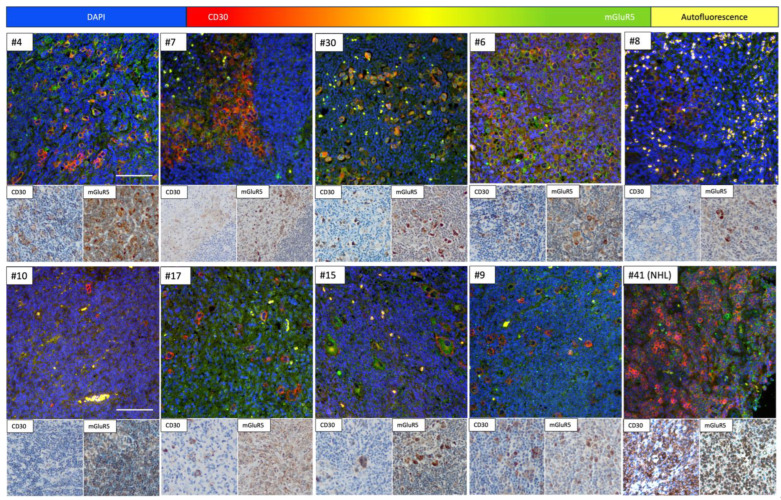
Examples of immunofluorescence co-staining with anti-CD30 and -mGluR5 antibodies on HL and NHL tissues with corresponding DAB staining images show mGluR5 expression in CD30+ tumor cells and examples of autofluorescent signal. Numbers #1-#30 belong to HL and #31-#62 to NHL samples. All HL sections except #10 and #17 show clearly double stained H-RS cells. The pattern for mGluR5 in #10 and #17 is more diffuse and no (elevated) expression is seen in CD30+ cells, both sections showed a DAB staining pattern for mGluR5 depicted in Figure 1b. #41 is an example of a CD30+ NHL (ALCL) with a high number of CD30+ cells and visible mGluR5 staining, which does not necessarily colocalize with CD30 in the IF images. Examples of autofluorescence are seen in #10 and #8 with multiple erythrocytes with excessive yellow staining due to exact overlap of red and green signal. The original objective lens magnification was 20× for all images, including DAB and IF stainings. The scale bar indicates 100 μm.

Given our group’s previous findings of heterogeneous *GRM5* expression in in vitro HL cell lines, the DAB staining results confirming the presence of mGluR5 in lymphoma tissues and the missing expression in healthy controls, we decided to further investigate the mGluR5 staining in HRS-cells [7]. We performed immunofluorescence co-staining of mGluR5 and CD30 in HL and CD30-positive NHL tissues, to better understand the localization and frequency of antigen expression. Examples are shown in Figure 2.

**Figure 3:**
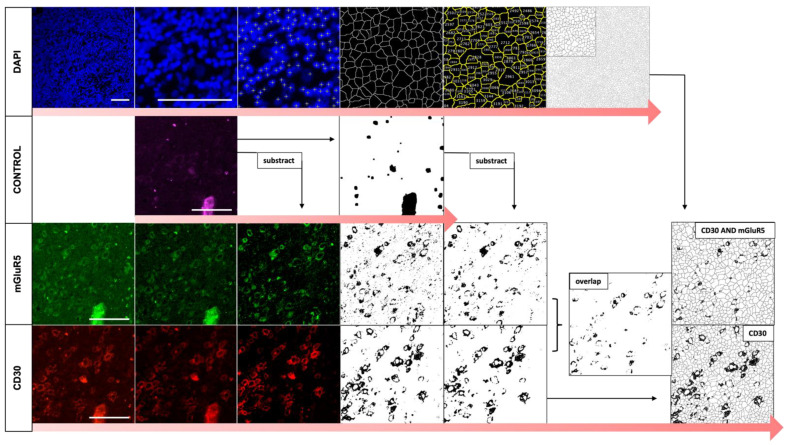
Image analysis of immunofluorescence co-staining: workflow visualization. The first row depicts the analysis of the total cell count by segmentation of the individual cell bodies using the DAPI stained nuclei. A standardized Gaussian blur filter was used to smooth out the intensities so that each nucleus could be automatically marked using the intensity maxima values. We then created a mask of the segmented nuclei, where each image pixel was assigned to a segment. We measured the created areas and counted each region < 500 µm^2^ as an independent cell. Each cell segment was saved as a ROI and given a serial number. The next rows show the process of thresholding for the creation of binary masks of the CD30 and mGluR5 stainings by subtracting background staining and autofluorescence recorded identified in control channel images. We created an overlap of the CD30 and mGluR5 binary masks using the AND parameter to visualize the areas of co-staining. The masks were then merged, and positive cell-segments (=including black pixels) were automatically counted. During this process we accepted the false-positive counting of neighboring cell bodies in case of staining overlap due to the uniformly used method. The scale bar indicates 100 μm.

The IF staining results were comparable to the DAB staining patterns and confirmed the presence of mGluR5 in CD30-positive H-RS cells of pediatric HL tissue. To quantify this finding, we analyzed the images for the total number of cells, the number of CD30-positive cells and the percentage of cells with both CD30 and mGluR5 positive signal, using an algorithm depicted in Figure 3. The result of absolute cell counts for all stained sections (n = 57) HL vs. NHL can be found in Supplemental Figure S2.

**Figure 4:**
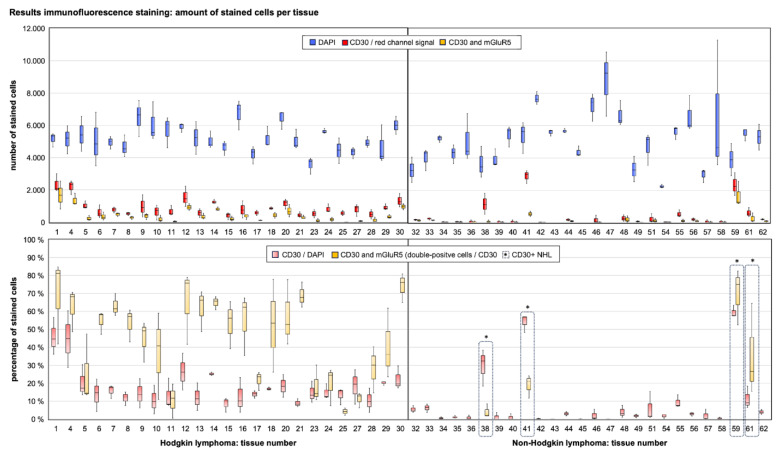
Heterogeneous numbers of CD30+ cells in HL and NHL tissues and expression of mGluR5 in CD30+ tumor cells. The x-axis shows the serial number of the tissue samples (#), where samples #1–30 belong to HL cases (left half) and samples #31–62 belong to NHL cases (right half). In the upper panel are the box plots depicting the image analysis results for absolute cell counts per tissue sample, showing varying amounts of CD30+ tumor cells independent from the total cell count. The box plots in the lower panel in light red depict the results in percent for CD30+ cells relative to the total cell count. In light yellow, the percentage of double positive (mGluR5 and CD30) tumor cells is shown relative to the total amount of CD30+ cells. For the NHL cases, the results are shown only for tissues with actual CD30-expression marked by an asterisk (*). Each box plot contains the results of n = 3 independent measurements of ×20 magnification images and the median is depicted as the center line.

**Figure 5:**
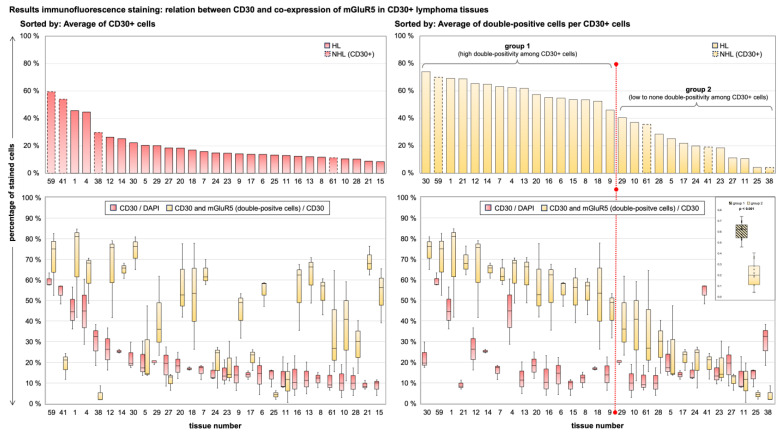
Sorting of image analysis results shows no correlation between CD30 and mGluR5 expression (in co-expressing lymphoma cells). We suggest the division into two groups of low and high mGluR5 expression in CD30+ tumor cells. The left panels of the graph show the mean results per tissue for the percentage of CD30+ cells and are sorted from highest to lowest. The right panels are sorted by the percentage of mGluR5 expression. All column plots show the mean of the measured parameter, the box plots include the individual measurements per tissue and indicate the minimum, maximum and median, as well as the 25th and 75th percentiles. The right panels include the 40% cut-off (red line) for dividing the subgroups. An inset box plot indicates a significant difference between the means of the two groups. Considering the highly sensitive image analysis algorithm, results of below 40% can be dismissed as low expression of the antigen. This corresponds with the DAB staining results.

To validate the specificity of the algorithm to detect CD30-positive cells, we compared the mean results per tissue for percentage of CD30-positive cells and saw significantly different values in CD30 expressing (n = 34) vs. CD30 negative (n = 23) sections (0.200 ± 0.136 vs. 0.032 ± 0.043; *p* < 0.001). For further analysis of the IF images, we excluded n = 6 (HL = 4, NHL = 2) tissue sections because of false positive analysis results for CD30 cell counts due to high background noise that could not be removed sufficiently by our standardized algorithm. Since the measurement of mGluR5 expression in H-RS cells via the algorithm is dependent on the accuracy of CD30-staining detection, we decided to exclude these images. Therefore, these sections do not take part in the subsequent calculations as well as Figure 4 and Figure 5.

The results for CD30/mGluR5 co-expression in HL and NHL cells are shown in Figure 4. The average of CD30-stained cells per tissue was 20.7% ± 13.4% with a range of 8.4–59.5% in CD30 positive tissues. The mean percentage of CD30-positive cells co-expressing mGluR5 was 43.0% ± 22.2% with a range of 4.1–74.0%. There was no significant difference between the HL and CD30-positive NHL groups for either parameter (Mann–Whitney U-Test: percentage of CD30+ cells *p* = 0.095; double positivity among CD30+ cells *p* = 0.408). When comparing the individual tumor cases, we found very heterogeneous expression of both antigens and no significant correlation between the mean values of the two parameters (Pearson r = 0.185, *p* = 0.335), indicating an independent expression of the antigens in lymphoma tumor cells.

To further compare the clinical data of our lymphoma cohort with our mGluR5 staining results, we sorted all samples by the average of double-positive tumor cells to create subgroups. We used the average of 40% as cut-off to define a high amount of double-positivity vs. low to no double-positivity. The difference in mean values per tissue between the two groups was significant (*p* < 0.001) as shown in Figure 5. This division was ultimately compatible with our DAB staining results, as all previously mentioned HL tissues with diffuse or homogeneous expression of mGluR5 without elevated expression in H-RS cells (Figure 1b) fell into the low to no double-positivity group. 

Next, we tested for a possible correlation between clinical parameters and histologic data with mGluR5 expression in CD30-positive cells, defined by the previously defined subgroups. These results are provided in Supplemental Table S1. There was no statistically significant correlation between for the parameters of sex, lymphoma subtype, onset of tumor-related symptoms and disease severity. To define disease severity, we used the following data: tumor staging, presence of B-symptoms, intensity of chemotherapy and use of ionizing radiation. We did not find any correlation with the incidence of relapse, the development of neurological or paraneoplastic symptoms or the recording of pathological EEG results.

We found significant results for the parameters histologic EBV-status and age at the time of diagnosis. The data suggest an inverse correlation between the percentage of mGluR5-positive H-RS cells and EBV-positive HL (*p* = 0.005). Second, our data indicate a positive correlation with age at diagnosis. Accordingly, the group with low percentage of mGluR5-positive H-RS cells was significantly younger at the time of diagnosis (*p* = 0.002) with a mean age of 11.5 years. The correlation factor (Pearson r for EBV association, Cramers V for age) indicated a moderate association for both parameters. The correlation can be considered as statistically significant, although a causality cannot be proven.

## 4. Discussion

Hodgkin lymphoma (HL) is a hematologic malignancy characterized by specific histologic features namely the Hodgkin–Reed–Sternberg (H-RS) cells and a distinct tumor microenvironment (TME) [16]. This malignancy shows a peak in children and young adults and accounts for the most common type of cancer in adolescents between 15–19 years of age [17]. HL is associated with the development of intermediate risk antibodies against the neuronal cell surface protein mGluR5 [18]. These antibodies have direct pathogenic potential, can cause autoimmune encephalitis and are 30–70% of cases associated with the presence of a tumor [13,18,19]. The molecular mechanism explaining the formation of paraneoplastic antibodies against cell surface antigens is still unknown [20]. A B-cell mediated process is thought to be responsible for the autoimmune response, in the sense of a systemic anticancer immune reaction, triggered by abnormally expressed neuronal antigens. Immunohistochemical data revealed relevant protein expression of antigens associated with paraneoplastic disease in respective cancer tissues, compatible with the hypothesis of the cancer itself as origin for the immunization process [21]. Recently, the first report of mGluR5 and *GRM5* expression in HL cell lines provided a first step towards understanding the nature of the association between mGluR5 antibody formation and Ophelia syndrome on a neurobiological level [8,22]. Now, in a consecutive histological and clinical investigation of a representative cohort of children and adolescents with HL, we have demonstrated the frequent presence of mGluR5 in HL tumor tissue and its lack of expression in healthy age-matched lymphatic tissues, as well as in non-malignant B lymphocytes of adults [23,24].

In most samples, we found elevated mGluR5 DAB and IF staining in H-RS cells. Only few samples showed homogeneous staining for mGluR5 in non-malignant inflammatory cells of the TME, without elevated signal in tumor cells. The findings are consistent with reports of glutamatergic signaling in the TME and differences in gene expression levels among H-RS cell populations [25,26]. We categorized the lymphoma cases into groups defined by high vs. low percentages of mGluR5-positive H-RS cells in order to explore clinical correlation with respect to disease severity.

Here, we discuss **(i)** the potential function of mGluR5 signaling in HL and **(ii)** possible implications for disease severity, including the observed association of mGluR5 expression with EBV status and age. In addition, we elaborate on **(iii)** the specificity of mGluR5 in HL and **(iv)** their potential pathogenicity for paraneoplastic neurological disease (PND) in pediatric lymphoma patients. 

**(i)** The physiological expression of mGluR5 has been described almost exclusively in the brain, where it is centrally involved in the regulation of postsynaptic membrane potential (for an overview of the physiological expression of mGluR5 and its associations with other genes, see Supplemental Figures S3 and S4). Aberrant regulation of mGluR5 signaling through postsynaptic proteins has been linked to the development of mental disorders, especially Schizophrenia and Autism Spectrum Disorder including the Fragile X Syndrome [3]. However, glutamatergic signaling is also known to drive tumor progression via autocrine and paracrine signaling that activates downstream pathways that are crucial for tumorigenesis [27,28,29]. Metabotropic receptors have been described as the most abundantly expressed glutamate signaling proteins in cancer [30]. Expression of mGluR5 has been reported in tumors of neuronal and non-neuronal origin, including lung cancer, glioma and renal cell carcinoma, among others [7,9,29,31]. mGluR5 in particular is described to support tumor progression and formation through activation of mitogen-activated protein kinase pathways (MAPK) via G protein-coupled induction of phospholipase C (PLC). The upregulation of mGluR5 protein levels in vivo led to the development of melanoma in transgenic mice. Further, it promoted in vitro tumor progression in multiple myeloma cells via enhanced MAPK signaling [10,12]. Osteosarcoma and hepatocellular carcinoma cells depend on mGluR5 for proliferation and survival by inhibition of apoptosis [11,32]. Previous research from our group linked mGluR5 upregulation in HL cell lines with increased expression of genes involved in MAPK and phosphoinositide-3-kinase (PI3K) signaling. [8]. A tumor-promoting function of mGluR5 in HL, consistent with cited knowledge about respective glutamatergic signaling in other cancers, seems possible [7,9,29,31]. 

**(ii)** A growing body of evidence implicates that variations in glutamate receptor expression may influence the course of malignant disease. In estrogen receptor (ER)-negative and triple-negative breast cancer, increased expression of the receptor mGluR1, comparable to mGluR5, is associated with a poor prognosis [33]. In oral squamous carcinoma, overexpression of mGluR5 correlates with improved overall survival [9]. Following our results of frequent detection of mGluR5 in H-RS cells and studies on histologic differences in HL acting as predictors of treatment response and outcome, we were interested in possible associations of disease parameters with mGluR5 expression in our HL cohort [24,34].

We did not find any correlation between mGluR5 expression and parameters indicating lymphoma aggressiveness or disease outcome. Given the size of our cohort and missing corresponding quantitative sequencing data, our results are not sufficient to confirm or reject such an association.

Interestingly, however, HL in adolescents and young adults (AYAs) has been described to present more aggressively with regard to staging and the factor extra-nodal and bulky disease as compared to HL in older adults and children below 14 years of age [35,36]. Our frequent finding of mGluR5 comes from a cohort of mostly adolescent HL patients above 15 years of age (18/29). Therefore, elevated mGluR5 expression could be specific to this population, potentially influencing the disease severity in this age group as HL samples of adult patients have not been studied. Reports on biological differences regarding mutational burden between H-RS cells of pediatric, AYA and adult HL with impact on clinical outcome support this hypothesis [35,37]. Fittingly, we observed a positive correlation between age at diagnosis and the percentage of H-RS cells expressing mGluR5. However, this association is most likely indirectly attributed to the inverse correlation between mGluR5 expression in H-RS cells and EBV-positive HL, as EBV-positive HL occurs more frequently in pediatric patients as compared to AYA cases, which also applied to our cohort [23,24,38]. 

In our cohort EBV-positivity was defined histologically by the positive staining for the viral oncoprotein LMP-1 [38]. In general, EBV-positive lymphoma is characterized by the presence of viral RNA and protein in tumor tissue. Positive EBV antibody titers in the blood of lymphoma patients alone as an indication of a prior or acute infection, do not automatically imply the presence of an EBV-positive tumor [39]. Our findings of an inverse correlation can be interpreted as an alignment with known molecular differences between EBV-positive and -negative HL [24,38]. It has been shown that EBV-positive H-RS cells have a significantly lower mutational load than EBV-negative cells [40]. Some authors have explained this with the oncogenic ability of viral proteins. LMP-1 specifically mimics activated TNF receptors causing induction of the NF-kB pathway that is crucial for tumor progression and survival in HL. Therefore, the presence of LMP-1 and other viral proteins in H-RS cells has been interpreted as a proxy for multiple tumor-promoting mutations otherwise “needed” in EBV-negative HL [39]. In general, H-RS cells depend on the constant activation of signaling pathways in order to escape apoptosis and detection by the immune system. In particular, due to a multitude of mutations and environmental factors the NF-kB, Pi3K, JAK-STAT and MAPK signaling pathways are over-activated and dysregulated [16,41]. In this context, our finding of higher mGluR5 expression on H-RS cells from EBV-negative tumors could be a hint to mGluR5 upregulation being one of many oncogenic alterations and tumor promoting factors described in the HL subtype. This hypothesis is generally supported by the previously described tumor-promoting effects of mGluR5 in other cancers, including the induction of MAPK signaling, which is also important for H-RS cell survival [12].

**(iii)** Another objective of our investigation was to determine whether mGluR5 expression was exclusively seen in Hodgkin lymphoma (HL) or would be present in other lymphoma entities as well, or even in tumor-free lymphatic tissue. Our results suggest that mGluR5 is equally present in the HL and NHL tissues of our cohort, including B cell and T cell malignancies, but appeared to be entirely absent in healthy lymphatic cells. We did not see mGluR5 in tumor-free or reactive lymph nodes and found no relevant expression of *GRM5* in RNA-sequencing data sets from isolated B and T lymphocyte populations. Interestingly, there was specifically no *GRM5*-expression in germinal center B cells, from which H-RS cells originally derive.

These findings are consistent with previously published reports about extra-neural mGluR5 expression in various cancers [42]. The fact that known oncogenic mutations of H-RS cells are also frequently found in other hematological malignancies fits with our finding of mGluR5 in different lymphoma subtypes—implying that we are pursuing the hypothesis of a tumor-promoting function of mGluR5 together with a potential role in the development of autoantibodies [16]. However, the question remains as to why the development of mGluR5 antibodies is specifically associated with HL, given the fact that the antigen is also present in other cancers. One explanation could be higher mGluR5 expression levels in lymphoma tissue as compared to other solid cancers. Frequent and strong antigen expression in an abnormal environment could increase the chances of triggering an immune response, namely the production of autoimmune antibodies, especially in an immunologically active environment such as lymph nodes [43]. This response may be additionally favored in lymphoma, as autoimmune diseases in general have been shown to increase the susceptibility to HL and NHL, indicating a generally more activated immune system in some lymphoma patients due to preexisting autoimmune diseases [44,45]. Still, the hypothesis of a causal relationship between mGluR5 antibodies and mGluR5 expression in lymphoma remains speculative and has not been proven.

**(iv)** Neurological symptoms are the most common type of paraneoplastic manifestation in HL [46]. Paraneoplastic cerebellar degeneration (PCD) and limbic encephalitis represent the most frequently observed forms of PND in HL patients [46,47]. Interestingly, both have been described to be associated with pathogenic antibodies against group I mGluRs—concerning cell surface antibody related disease [48]. Both PCD and limbic encephalitis in HL (Ophelia Syndrome) are associated with mGluR5 antibodies [49]. A deeper functional connection between HL and mGluRs remains to be elucidated, as no other cancer entity has been so frequently associated with antibodies against these receptors.

In recent years, the number of reported cases of PND have risen significantly, possibly due to increased awareness among clinicians [50]. As PND is still considered to be rare in HL—if compared to other cancers—we were interested in whether characteristic symptoms were being missed in this patient cohort or whether mild phenotypes had been overlooked [22]. Our search did not reveal any patients with neurological symptoms meeting the diagnostic criteria for PND, except for the two patients already reported before (Ophelia syndrome, depression) [18]. However, we did find three additional cases with unexplained neurological abnormalities (another case of depression, one case of fever and psychosis, one abnormal EEG with epileptic discharges) at the time of the cancer diagnosis. Taken together, given the small size of our cohort and the analysis of only retrospective data, this is a high prevalence. PND is generally thought to occur more frequently in HL rather than in NHL and interestingly, we did not find any NHL patients with either paraneoplastic disease or unexplained neurological symptoms.

### Limitations

The main limitation of our study was the size of our cohort. Since the majority of lymphoma tissue samples were not available for research at our pathology institute or at the respective reference centers, we were only able to study mGluR5 in less than half of the pediatric HL patients diagnosed in recent years. It is important to consider that our cohort included only patients under 18 years of age who had been treated by pediatric oncologists. Previously reported differences between adult, pediatric and AYA HL may also apply to mGluR5 expression, and therefore our results cannot be generalized [34,36]. 

## 5. Conclusions

The results presented are compatible with the hypothesis of a possible causal relationship between mGluR5 antibody formation and Ophelia syndrome. We present a novel piece of information as we demonstrated frequent expression of mGluR5 in HL tissues of pediatric and AYA patients, while showing a lack of expression in healthy age-matched lymph node tissue as well as normal B and T cells. A tumor-directed autoimmune response could be triggered by the mGluR5 expression in malignant cells. Previous data on mGluR5 in H-RS cells and metabotropic glutamatergic signaling in cancer supporting tumor progression, are consistent with our findings on mGluR5 in HL and NHL. The observed correlation between mGluR5 staining and EBV status of HL tissues along with age in our cohort and previous research on glutamate signaling can be interpreted as a first indication of an oncogenic function of mGluR5 in HL, which needs to be elaborated in the future. The differential expression of mGluR5 and its potential impact on disease presentation may be in line with reported molecular differences between adult, pediatric and AYA HL. Therefore, further investigations including all age groups and a larger cohort of HL patients are needed to substantiate the suspected correlations with outcome, severity and especially neurologic symptoms. Regarding the latter, independent of our findings on mGluR5, we observed a disequilibrium in the prevalence of paraneoplastic neurological symptoms between HL and NHL patients. Therefore, it is important to consider the possibility of under-recognition of PND or the presence of mild phenotypes, especially in patients with HL. In conclusion, future studies should assess the potential of mGluR5 as an additional prognostic or diagnostic marker for lymphoma.

## Figures and Tables

**Table 1:** Analysis of patient and disease characteristics: comparison between the HL and NHL cohorts. The total number of cases per cohort is provided in the first row. The parameters are depicted in absolute numbers. Relative numbers in brackets refer to the total of the respective cohort.

	HL		NHL	
	n = 29		n = 28	
**Sex**				
female	17/29	(59%)	3/28	(11%)
male	12/29	(41%)	25/28	(89%)
Age *				
mean, [range]	14.1	[5.5–17.5]	10.5	[3.0–17.8]
Staging **				
I	0/29	(0%)	2/28	(7%)
II	17/29	(59%)	8/28	(29%)
III	4/29	(14%)	9/28	(32%)
IV	8/29	(27%)	9/28	(32%)
B-symptoms	11/29	(38%)	5/28	(18%)
Onset of symptoms ***				
mean, [range]	4.7	[1–27]	1.9	[0.25–9]
**Event free survival status**				
0—no event	25/29	(86%)	23/28	(82%)
1—event (relapse)	4/29	(14%)	3/28	(11%)
no data	0/29	(0%)	2/28	(7%)
**Overall survival status**				
0—alive	28/29	(97%)	26/28	(92%)
1—succumbed to disease	1/29	(3%)	1/28	(4%)
2—died of other causes	0/29	(0%)	1/28	(4%)
**Special symptoms**				
neurological-psychiatric	4/29	(14%)	6/28	(21%)
CNS involvement	0/4	(0%)	6/6	(100%)
paraneoplastic	3/29	(10%)	0/28	(0%)
**Neurological diagnostics**				
EEG	21/29	(72%)	19/28	(68%)
cMRI	8/29	(27%)	11/28	(39%)
CSF	5/29	(17%)	28/28	(100%)
antineuronal antibody screening	2/29	(6%)	0/28	(0%)

* Age is presented as mean and total range of years at the time of diagnosis. ** Staging data refer to the Ann Arbor classification for HL and the St. Jude’s classification for NHL. *** Onset of symptoms describes the duration in months from the first recorded lymphoma related symptom to diagnosis. CNS involvement = central nervous system involvement of the lymphoma (diagnosed by CSF and/or cMRI); EEG = electroencephalography; cMRI = cerebral MRI; CSF = cerebral spinal fluid analysis.

## Data Availability

The raw data supporting the conclusions of this article will be made available by the authors on request.

## Supplementary Materials

The following supporting information can be downloaded at: https://www.mdpi.com/article/10.3390/cancers16132452/s1, Figure S1: Distribution of lymphoma subtypes per cohort, Figure S2: Results for IF image analysis: absolute cell count numbers; Table S1: Statistical analysis results; Code for ImageJ/Fiji Macro for automated image analysis. Figure S3: Gene expression data for GRM5, the gene encoding for mGluR5. Figure S4: Physiological function of mGluR5 illustrated by its predicted protein-protein interaction network.

## References

[1] Carr I. (1982). The Ophelia syndrome: Memory loss in Hodgkin’s disease. Lancet.

[2] Lancaster E., Martinez-Hernandez E., Titulaer M., Boulos M., Weaver S., Antoine J.-C., Liebers E., Kornblum C., Bien C., Honnorat J. (2011). Antibodies to metabotropic glutamate receptor 5 in the Ophelia syndrome. Neurology.

[3] Niswender C.M., Conn P.J. (2010). Metabotropic glutamate receptors: Physiology, pharmacology, and disease. Annu. Rev. Pharmacol. Toxicol..

[4] Levite M. (2014). Glutamate receptor antibodies in neurological diseases: Anti-AMPA-GluR3 antibodies, anti-NMDA-NR1 antibodies, anti-NMDA-NR2A/B antibodies, anti-mGluR1 antibodies or anti-mGluR5 antibodies are present in subpopulations of patients with either: Epilepsy, encephalitis, cerebellar ataxia, systemic lupus erythematosus (SLE) and neuropsychiatric SLE, Sjogren’s syndrome, schizophrenia, mania or stroke. These autoimmune anti-glutamate receptor antibodies can bind neurons in few brain regions, activate glutamate receptors, decrease glutamate receptor’s expression, impair glutamate-induced signaling and function, activate blood brain barrier endothelial cells, kill neurons, damage the brain, induce behavioral/psychiatric/cognitive abnormalities and ataxia in animal models, and can be removed or silenced in some patients by immunotherapy. J. Neural Transm..

[5] Ruiz-Garcia R., Martínez-Hernández E., Joubert B., Petit-Pedrol M., Pajarón-Boix E., Fernández V., Salais L., del Pozo M., Armangué T., Sabater L. (2020). Paraneoplastic cerebellar ataxia and antibodies to metabotropic glutamate receptor 2. Neurol. Neuroimmunol. Neuroinflamm..

[6] Sillevis Smitt P., Kinoshita A., De Leeuw B., Moll W., Coesmans M., Jaarsma D., Henzen-Logmans S., Vecht C., De Zeeuw C., Sekiyama N. (2000). Paraneoplastic cerebellar ataxia due to autoantibodies against a glutamate receptor. N. Engl. J. Med..

[7] Brocke K.S., Staufner C., Luksch H., Geiger K.D., Stepulak A., Marzahn J., Schackert G., Temme A., Ikonomidou C. (2010). Glutamate receptors in pediatric tumors of the central nervous system. Cancer Biol. Ther..

[8] Schnell S., Knierim E., Bittigau P., Kreye J., Hauptmann K., Hundsdoerfer P., Morales-Gonzalez S., Schuelke M., Nikolaus M. (2023). Hodgkin Lymphoma Cell Lines and Tissues Express mGluR5: A Potential Link to Ophelia Syndrome and Paraneoplastic Neurological Disease. Cells.

[9] Park S.Y., Lee S.-A., Han I.-H., Yoo B.-C., Lee S.-H., Park J.-Y., Cha I.-H., Kim J., Choi S.-W. (2007). Clinical significance of metabotropic glutamate receptor 5 expression in oral squamous cell carcinoma. Oncol. Rep..

[10] Choi K.Y., Chang K., Pickel J.M., Badger J.D., Roche K.W. (2011). Expression of the metabotropic glutamate receptor 5 (mGluR5) induces melanoma in transgenic mice. Proc. Natl. Acad. Sci. USA.

[11] Liao S., Ruiz Y., Gulzar H., Yelskaya Z., Taouit L.A., Houssou M., Jaikaran T., Schvarts Y., Kozlitina K., Basu-Roy U. (2017). Osteosarcoma cell proliferation and survival requires mGluR5 receptor activity and is blocked by Riluzole. PLoS ONE.

[12] Kou W., Huang H., Dai S., Tan X., Chen Q., Huang R., Zou H. (2022). mGluR5 promotes the progression of multiple myeloma in vitro via Ras-MAPK signaling pathway. Adv. Clin. Exp. Med..

[13] Maudes E., Mannara F., García-Serra A., Radosevic M., Mellado A., Serafim A.B., Planagumà J., Sabater L., Dalmau J., Spatola M. (2022). Human Metabotropic Glutamate Receptor 5 Antibodies Alter Receptor Levels and Behavior in Mice. Ann. Neurol..

[14] Spatola M., Sabater L., Planagumà J., Martínez-Hernandez E., Armangué T., Prüss H., Iizuka T., Oblitas R.L.C., Antoine J.-C., Li R. (2018). Encephalitis with mGluR5 antibodies: Symptoms and antibody effects. Neurology.

[15] Buchwalow I., Atiakshin D., Samoilova V., Boecker W., Tiemann M. (2018). Identification of autofluorescent cells in human angioimmunoblastic T-cell lymphoma. Histochem. Cell Biol..

[16] Weniger M.A., Kuppers R. (2021). Molecular biology of Hodgkin lymphoma. Leukemia.

[17] Flerlage J.E., Hiniker S.M., Armenian S., Benya E.C., Bobbey A.J., Chang V., Cooper S., Coulter D.W., Cuglievan B., Hoppe B.S. (2021). Pediatric Hodgkin Lymphoma, Version 3. 2021. J. Natl. Compr. Cancer Netw..

[18] Graus F., Vogrig A., Muñiz-Castrillo S., Antoine J.-C.G., Desestret V., Dubey D., Giometto B., Irani S.R., Joubert B., Leypoldt F. (2021). Updated Diagnostic Criteria for Paraneoplastic Neurologic Syndromes. Neurol. Neuroimmunol. Neuroinflamm..

[19] Dalmau J., Geis C., Graus F. (2017). Autoantibodies to Synaptic Receptors and Neuronal Cell Surface Proteins in Autoimmune Diseases of the Central Nervous System. Physiol. Rev..

[20] Marsili L., Marcucci S., LaPorta J., Chirra M., Espay A.J., Colosimo C. (2023). Paraneoplastic Neurological Syndromes of the Central Nervous System: Pathophysiology, Diagnosis, and Treatment. Biomedicines.

[21] Binks S., Uy C., Honnorat J., Irani S.R. (2022). Paraneoplastic neurological syndromes: A practical approach to diagnosis and management. Pract. Neurol..

[22] Graus F., Arino H., Dalmau J. (2014). Paraneoplastic neurological syndromes in Hodgkin and non-Hodgkin lymphomas. Blood.

[23] Punnett A., Tsang R.W., Hodgson D.C. (2010). Hodgkin lymphoma across the age spectrum: Epidemiology, therapy, and late effects. Semin. Radiat. Oncol..

[24] Connors J.M., Cozen W., Steidl C., Carbone A., Hoppe R.T., Flechtner H.H., Bartlett N.L. (2020). Hodgkin lymphoma. Nat. Rev. Dis. Primers.

[25] Koda S., Hu J., Ju X., Sun G., Shao S., Tang R.-X., Zheng K.-Y., Yan J. (2023). The role of glutamate receptors in the regulation of the tumor microenvironment. Front. Immunol..

[26] Pacheco R., Ciruela F., Casadó V., Mallol J., Gallart T., Lluis C., Franco R. (2004). Group I metabotropic glutamate receptors mediate a dual role of glutamate in T cell activation. J. Biol. Chem..

[27] Prickett T.D., Samuels Y. (2012). Molecular pathways: Dysregulated glutamatergic signaling pathways in cancer. Clin. Cancer Res..

[28] Willard S.S., Koochekpour S. (2013). Glutamate signaling in benign and malignant disorders: Current status, future perspectives, and therapeutic implications. Int. J. Biol. Sci..

[29] Yu L.J., Wall B.A., Wangari-Talbot J., Chen S. (2017). Metabotropic glutamate receptors in cancer. Neuropharmacology.

[30] Liu X., Zhang Y., Wang Z., Wang X., Zhu G., Han G., Chen G., Hou C., Wang T., Shen B. (2016). Metabotropic glutamate receptor 3 is involved in B-cell-related tumor apoptosis. Int. J. Oncol..

[31] Huang C.Y., Hsueh Y., Chen L., Cheng W., Yu C., Chen W., Lu T., Lan K., Lee C., Huang S. (2018). Clinical significance of glutamate metabotropic receptors in renal cell carcinoma risk and survival. Cancer Med..

[32] Bai X.X., Gu L., Yang H.M., Xi S.S., Xia N., Zhang S., Zhang H. (2018). Downregulation of metabotropic glutamate receptor 5 inhibits hepatoma development in a neurotoxin rotenone-induced Parkinson’s disease model. Toxicol. Lett..

[33] Bastiaansen A.E.M., Timmermans A.M., Smid M., van Deurzen C.H.M., Hulsenboom E.S.P., der Smissen W.J.C.P.-V., Foekens R., Trapman-Jansen A.M.A.C., Smitt P.A.E.S., Luider T.M. (2020). Metabotropic glutamate receptor 1 is associated with unfavorable prognosis in ER-negative and triple-negative breast cancer. Sci. Rep..

[34] Chetaille B., Bertucci F., Finetti P., Esterni B., Stamatoullas A., Picquenot J.M., Copin M.C., Morschhauser F., Casasnovas O., Petrella T. (2009). Molecular profiling of classical Hodgkin lymphoma tissues uncovers variations in the tumor microenvironment and correlations with EBV infection and outcome. Blood.

[35] Metzger M.L., Mauz-Korholz C. (2019). Epidemiology, outcome, targeted agents and immunotherapy in adolescent and young adult non-Hodgkin and Hodgkin lymphoma. Br. J. Haematol..

[36] Bigenwald C., Galimard J.-E., Quero L., Cabannes-Hamy A., Thieblemont C., Boissel N., Brice P. (2017). Hodgkin lymphoma in adolescent and young adults: Insights from an adult tertiary single-center cohort of 349 patients. Oncotarget.

[37] Maura F., Ziccheddu B., Xiang J.Z., Bhinder B., Rosiene J., Abascal F., Maclachlan K.H., Eng K.W., Uppal M., He F. (2023). Molecular Evolution of Classic Hodgkin Lymphoma Revealed Through Whole-Genome Sequencing of Hodgkiand Reed Sternberg Cells. Blood Cancer Discov..

[38] Massini G., Siemer D., Hohaus S. (2009). EBV in Hodgkin Lymphoma. Mediterr. J. Hematol. Infect. Dis..

[39] Vockerodt M., Yap L., Shannon-Lowe C., Curley H., Wei W., Vrzalikova K., Murray P.G. (2015). The Epstein-Barr virus and the pathogenesis of lymphoma. J. Pathol..

[40] Wienand K., Chapuy B., Stewart C., Dunford A.J., Wu D., Kim J., Kamburov A., Wood T.R., Cader F.Z., Ducar M.D. (2019). Genomic analyses of flow-sorted Hodgkin Reed-Sternberg cells reveal complementary mechanisms of immune evasion. Blood Adv..

[41] Weniger M.A., Kuppers R. (2016). NF-kappaB deregulation in Hodgkin lymphoma. Semin. Cancer Biol..

[42] Stepulak A., Luksch H., Gebhardt C., Uckermann O., Marzahn J., Sifringer M., Rzeski W., Staufner C., Brocke K.S., Turski L. (2009). Expression of glutamate receptor subunits in human cancers. Histochem. Cell Biol..

[43] Johnson L.A. (2021). In Sickness and in Health: The Immunological Roles of the Lymphatic System. Int. J. Mol. Sci..

[44] Masciopinto P., Dell’olio G., De Robertis R., Specchia G., Musto P., Albano F. (2020). The Role of Autoimmune Diseases in the Prognosis of Lymphoma. J. Clin. Med..

[45] Kristinsson S.Y., Landgren O., Sjöberg J., Turesson I., Björkholm M., Goldin L.R. (2009). Autoimmunity and risk for Hodgkin’s lymphoma by subtype. Haematologica.

[46] El Fakih R., Bajuaifer Y.S., Shah A.Y., Sulaiman R., Almohamady R., Elgohary G., Alothaimeen H.S., Aljurf M. (2024). Paraneoplastic syndromes associated with classic Hodgkin lymphoma, a systematic literature review. Ann. Hematol..

[47] Briani C., Vitaliani R., Grisold W., Honnorat J., Graus F., Antoine J., Bertolini G., Giometto B., Euronetwork F.T.P. (2011). Spectrum of paraneoplastic disease associated with lymphoma. Neurology.

[48] Scotton W.J., Karim A., Jacob S. (2019). Glutamate Receptor Antibodies in Autoimmune Central Nervous System Disease: Basic Mechanisms, Clinical Features, and Antibody Detection. Methods Mol. Biol..

[49] Lancaster E. (2017). CNS syndromes associated with antibodies against metabotropic receptors. Curr. Opin. Neurol..

[50] Vogrig A., Gigli G.L., Segatti S., Corazza E., Marini A., Bernardini A., Valent F., Fabris M., Curcio F., Brigo F. (2020). Epidemiology of paraneoplastic neurological syndromes: A population-based study. J. Neurol..

