# Supplementary material for: Expression of mGluR5 in Pediatric Hodgkin and Non-Hodgkin lymphoma—A Comparative Analysis of Immunohistochemical and Clinical Findings Regarding the Association between Tumor and Paraneoplastic Neurological Disease
Source: Cancers (Basel). 2024 Jul 4;16(13):2452. doi: 10.3390/cancers16132452 (PMC11240514; doi:10.3390/cancers16132452)
Supplement: Supplementary file 1 [file cancers-16-02452-s001.zip › cancers-3028718-supplementary.pdf]

Supplemental Material:

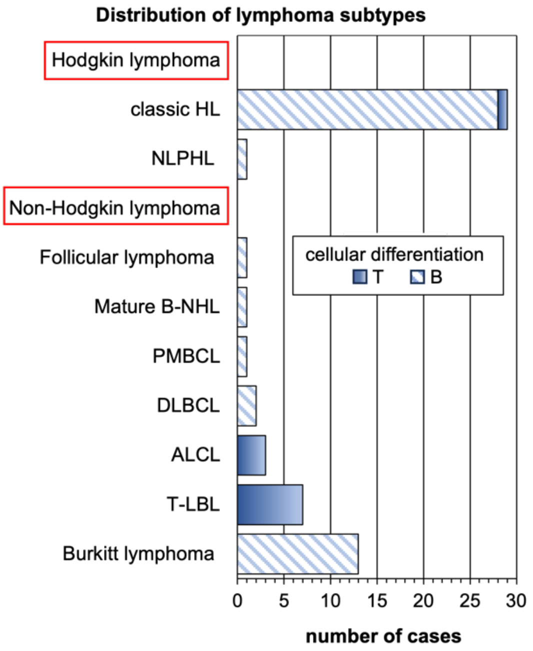

Figure S1: The absolute numbers of the different histologic subtypes present in our cohort of HL and NHL cases. The coloring of the columns represents B- and T-cell lymphomas. The case of mature B-NHL was not further classified. Abbreviations: Nodular lymphocyte-predominant HL (NLPHL), Primal mediastinal B-cell lymphoma (PMBCL), Diffuse large B-cell lymphoma (DLBCL), Anaplastic large-cell lymphoma (ALCL), T-Lymphoblastic lymphoma (T-LBL).

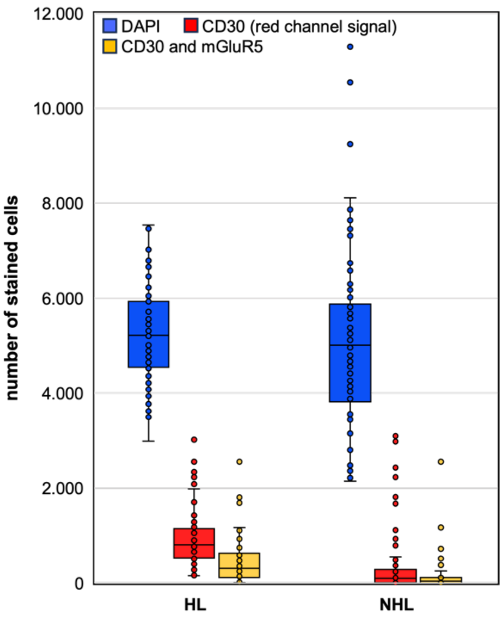

Figure S2: Image analysis results for absolute cell counts in all tissue samples (n=57). individual results for overall total cell counts are shown in blue (all DAPI-stained cells), CD30+ cells in red, and co-stained mGluR5 and CD30+ cells in yellow. The box plots include the mean results of each tissue sample and indicate the median. Our results show comparable numbers of cells per sample with a higher variability in NHL cases. Since the NHL cohort contains only a few cases with documented CD30+ cells, the median result for this parameter is significantly lower. The outliers with numbers comparable to the HL results for CD30 belong to the CD30+ NHL cases and a few cases with false positive results.

Amount of mGluR5+ cilles among CD30+ cells – high vs low to none	HL (n=25)			
**Nominal and ordinal variables: Chi-sqaure or Fishers exact test**				
**Variable/ parameter**	**x^2(1)**	**Chi-square: p-value**	**Fishers: p-value**	**Cramers V**
**age** (<15; ≥ 15 years)	6,84	**0,009**	**0,015**	**0,523**
**sex** (male;female)	0,244	0,622	0,697	
**histological HL subtype** (NS; MT)	4,001	0,045	0,075	
**histological association to EBV** (yes; no)	9,375	**0,002**	**0,005**	**0,612**
**EBV serology** (positive; negative)	4,890	**0,027**	**0,04**	**0,442**
**B-symptoms** (yes; no)	0,115	0,735	1,0	
**radiation** (yes; no)	1,042	0,307	0,358	
**staging** (I;II;III;IV)	0,278	0,870		
**therapy arm for chemotherapy** (mild; intermediate; advanced)	0,069	0,996		
**relapse (**yes; no)	1,449	0,229	0,5	
**neurological symptoms** (yes; no)	0,063	0,802	1,0	
**pathological EEG** (yes; no)	0,063	0,802	1,0	
**paraneoplastic symptoms** (yes; no)	0,091	0,763	1,0	
**Metric variables: Mann-Whitney-U test**				
**Variable/ parameter**	**U**	**p-value**	**Z**	**r**
**onset of symptoms** (months before diagnosis)	56	0,311		
**age** (at time of diagnosis)	22	**0,002**	-2,943	0,59

Table S1: Statistical analysis. Nominal and ordinal variables are shown at the top, the metric variables at the bottom. The test for significant difference of the mean values between the individual clinical parameters and the groups defined by the percentage of mGluR5+ H-RS cells (Figure 5) was calculated. All statistically significant results are highlighted.

Code for ImageJ/Fiji Macro for automated image analysis:

https://github.com/vieing/githubpage_sup_materials/blob/e7d2134bc31c12c3aec328a03488bb716cc40d36/Code%20for%20analysis%20of%20IF%20images_Ophelia%20syndrome%20project.ijm

Physiological mGluR5 gene expression and predicted function

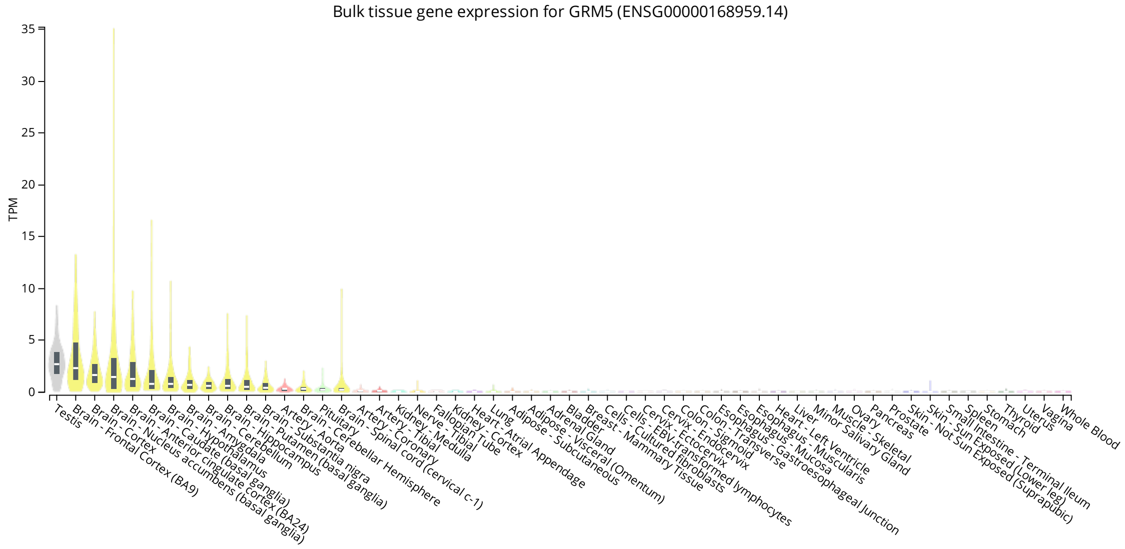

Figure S3: Gene expression data for *GRM5*, the gene encoding for mGluR5. *GRM5* is predominantly expressed in brain and testis. Gene expression in other tissues and cell types is almost negligible, in particular no expression is reported for lymphatic tissues or white blood cells, which is consistent with our immunohistologic and RNA-seq data. Data Source: GTEx Analysis Release V8 (dbGaP Accession phs000424.v8.p2) https://gtexportal.org/home/gene/GRM5#geneExpression

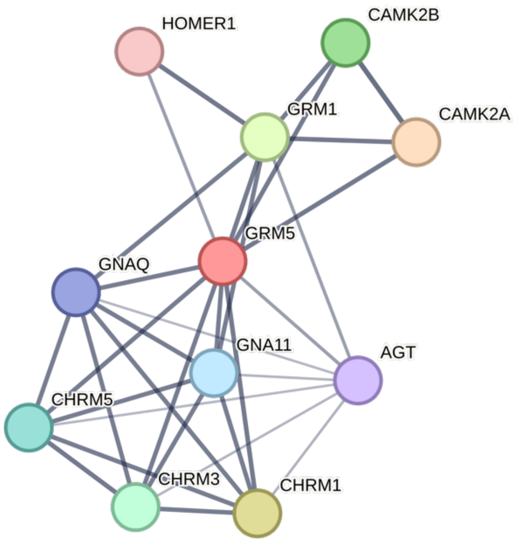

Figure S4: Physiological function of mGluR5 illustrated by its predicted protein-protein interaction network. The *GRM5* encoded mGluR5 is as G protein-coupled receptor predominantly expressed at the postsynaptic sites of human neurons. It is involved in the regulation of postsynaptic membrane potential, by controlling cytosolic calcium ion concentration and activating a variety of central cell signaling pathways including other G proteins, phospholipase c, and Homer1 - exemplary list below.

CAMK2A	Calcium/calmodulin-dependent protein kinase type II subunit alpha; is involved in synaptic plasticity, neurotransmitter release and long-term potentiation. Member of the NMDAR signaling complex in excitatory synapses.
CHRM1	Muscarinic acetylcholine receptor M1; mediates various cellular responses, including inhibition of adenylate cyclase, breakdown of phosphoinositides and modulation of potassium channels through the action of G proteins.
GRM1	Metabotropic glutamate receptor 1; Signaling activates a phosphatidylinositol-calcium second messenger system. May participate in the central action of glutamate in the CNS, such as long-term potentiation in the hippocampus and long-term depression in the cerebellum.
CAMK2B	Calcium/calmodulin-dependent protein kinase type II subunit beta; involved in dendritic spine and synapse formation, neuronal plasticity and regulation of sarcoplasmic reticulum Ca(2+) transport in skeletal muscle.
CHRM3	Muscarinic acetylcholine receptor M3; see M1
CHRM5	Muscarinic acetylcholine receptor M5; see M1
GNA11	Guanine nucleotide-binding protein subunit alpha-11; involved as modulators or transducers in various transmembrane signaling systems. Acts as an activator of phospholipase C.
GNAQ	Guanine nucleotide-binding protein G(q) subunit alpha; involved as modulators or transducers in various transmembrane signaling systems.
AGT	Angiotensin; Essential component of the renin-angiotensin system (RAS).
HOMER1	Homer protein homolog 1; Postsynaptic density scaffolding protein. Binds and cross- links cytoplasmic regions of GRM1, GRM5, ITPR1, DNM3, RYR1, RYR2, SHANK1 and SHANK3. By physically linking GRM1 and GRM5 with ER-associated ITPR1 receptors, it aids the coupling of surface receptors to intracellular calcium release.

Data Source: https://version-12-0.string-db.org/cgi/network?networkId=bq7yZNwUUQbK
